# Supplementary material for: ALKBH7 mediates necrosis via rewiring of glyoxal metabolism
Source: eLife. 2020 Aug 14;9:e58573. doi: 10.7554/eLife.58573 (PMC7442491; doi:10.7554/eLife.58573)
Supplement: Supplementary file 1. — Cells transfected with FLAG-tagged ALKBH7 were treated with MMS as per the methods, the anti-FLAG beads used to immunoprecipitate ALKBH7-interacting proteins. Following SDS-PAGE separation (see Figure 2—figure supplement 1), excised bands were identified by mass spectrometry of trypsin digests. Proteins are listed by name and accession, with the number of unique peptides identified under each condition indicated in the appropriate columns. [file elife-58573-supp1.docx]

| *Protein* | *Accession* | *# peptides*  *Control* | *# peptides*  *MMS-treated* |
| --- | --- | --- | --- |
| AlkB homolog 7 | ALKB7 | 9 | 9 |
| 60 kDa heat shock protein, mitochondrial | CH60 | 3 | 10 |
| Heat shock cognate 71 kDa protein | HSP7C | 6 | 1 |
| Heat shock 70 kDa protein | HSP76 | 3 | 2 |
| T-complex protein 1 subunit ε | TCPE | 1 | 2 |
| cDNA FLJ53752, highly similar to Heat shock 70 kDa protein 1 | B4DNX1 | 2 | 0 |
| cDNA FLJ56386, highly similar to Heat shock 70 kDa protein 1L | B4DI54 | 3 | 0 |
| 10 kDa heat shock protein, mitochondrial | CH10 | 0 | 1 |
| UPF0693 protein C10orf32 | CJ032 | 0 | 1 |
| A-kinase anchor protein 12 | AKA12 | 1 | 0 |
| cDNA FLJ56389, highly similar to Elongation factor 1 γ | B4DTG2 | 1 | 0 |
| Heat shock 70 kDa protein 4 | HSP74 | 1 | 0 |
| Heat shock 70kDa protein 1A variant | Q59EJ3 | 1 | 0 |
| cDNA clone CS0DF029YN24 of Fetal brain | Q86U40 | 1 | 0 |
| RL40_HUMAN (P62987) Ubiquitin-60S ribosomal protein L40 | RL40 | 1 | 0 |
| 52 kDa Ro protein | RO52 | 1 | 0 |
| RuvB-like 1 | RUVB1 | 1 | 0 |
| Elongation factor 1-β | EF1B | 0 | 1 |
| Hemoglobin subunit β | HBB | 0 | 1 |
| NADH dehydrogenase iron-sulfur protein 7, mitochondrial | NDUS7 | 0 | 1 |
| Tubulin β-1 chain | TBB1 | 0 | 1 |
| KLRAQ motif-containing protein 1 | KLRAQ | 0 | 1 |
| cDNA FLJ36455, clone THYMU2014323, highly similar to Stabilin-1 | B3KSK0 | 0 | 1 |

**Supplementary Table 1. Proteins identified as interacting with ALKBH7 by immunoprecipitation under control or MMS-treated conditions.** Cells transfected with FLAG-tagged ALKBH7 were treated with MMS as per the methods, the anti-FLAG beads used to immunoprecipitate ALKBH7-interacting proteins. Following SDS-PAGE separation (see Fig. S3), excised bands were identified by mass spectrometry of trypsin digests. Proteins are listed by name and accession, with the number of unique peptides identified under each condition indicated in the appropriate columns.
